# Mesothelin, Stereocilin, and Otoancorin are predicted to have superhelical structures with ARM-type repeats

**DOI:** 10.1186/1472-6807-9-1

**Published:** 2009-01-07

**Authors:** Bangalore K Sathyanarayana, Yoonsoo Hahn, Manish S Patankar, Ira Pastan, Byungkook Lee

**Affiliations:** 1 Laboratory of Molecular Biology, Center for Cancer Research, National Cancer Institute, NIH, Bethesda, Maryland 20892-4264, USA; 2 Department of Life Science, College of Natural Science, Chung-Ang University, Seoul 156-756, South Korea; 3 Department of Obstetrics and Gynecology, University of Wisconsin-Madison, Madison, WI, USA

## Abstract

**Background:**

Mesothelin is a 40 kDa protein present on the surface of normal mesothelial cells and overexpressed in many human tumours, including mesothelioma and ovarian and pancreatic adenocarcinoma. It forms a strong and specific complex with MUC16, which is also highly expressed on the surface of mesothelioma and ovarian cancer cells. This binding has been suggested to be the basis of ovarian cancer metastasis. Knowledge of the structure of this protein will be useful, for example, in building a structural model of the MUC16-mesothelin complex. Mesothelin is produced as a precursor, which is cleaved by furin to produce the N-terminal half, which is called the megakaryocyte potentiating factor (MPF), and the C-terminal half, which is mesothelin. Little is known about the function of mesothelin and there is no information on its possible three-dimensional structure. Mesothelin has been reported to be homologous to the deafness-related inner ear proteins otoancorin and stereocilin, for neither of which the three-dimensional structure is known.

**Results:**

The BLAST and PSI-BLAST searches confirmed that mesothelin and mesothelin precursor proteins are remotely homologous to stereocilin and otoancorin and more closely homologous to the hypothetical protein MPFL (MPF-like). Secondary structure prediction servers predicted a predominantly helical structure for both mesothelin and mesothelin precursor proteins and also for stereocilin and otoancorin. Three-dimensional structure prediction servers INHUB and I-TASSER produced structural models for mesothelin, which consisted of superhelical structures with ARM-type repeats in conformity with the secondary structure predictions. Similar ARM-type superhelical repeat structures were predicted by 3D-PSSM server for mesothelin precursor and for stereocilin and otoancorin proteins.

**Conclusion:**

The mesothelin superfamily of proteins, which includes mesothelin, mesothelin precursor, megakaryocyte potentiating factor, MPFL, stereocilin and otoancorin, are predicted to have superhelical structures with ARM-type repeats. We suggest that all of these function as superhelical lectins to bind the carbohydrate moieties of extracellular glycoproteins.

## Background

**Figure 1:**
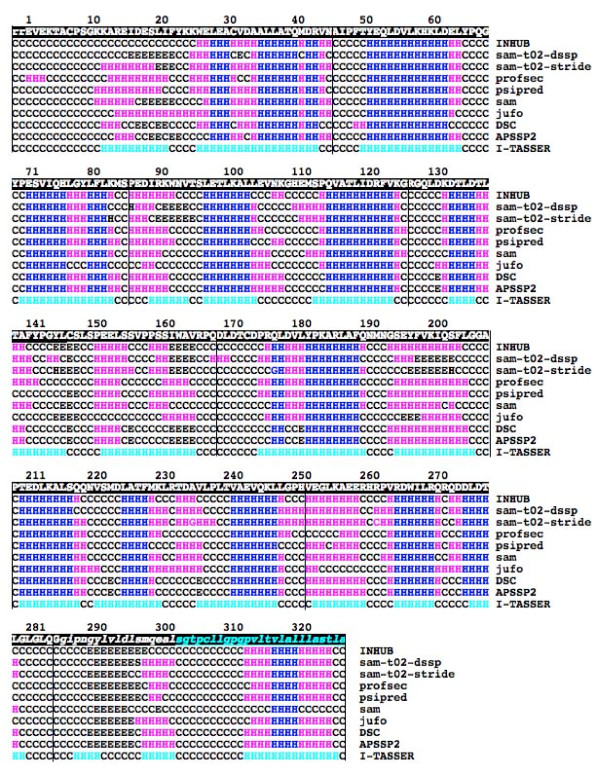
**Amino acid sequence and predicted secondary structure for human mesothelin**. The first line gives the residue serial numbers and the second the sequence. The mature mesothelin sequence starts from the residue number 1, after the furin cleavage at the ARG-ARG (rr) sequence of the precursor. The signalling sequence for the GPI attachment that was suggested in an earlier study [1] is shown in low-case italics; the signalling sequence predicted using a current prediction program [45] is colored green. The 9 lines that follow give the predicted secondary structural type for each residue. The names of the programs are indicated at the right-hand side. The blue color indicates residues that were predicted to be helical unanimously by all programs. The magenta indicates all other helical predictions. The last line gives the secondary structures of model#1 of the I-TASSER server, calculated using the DSSP program (H: Helix, program output states H, h, G; E: Beta, E; C: Coil, all other output states). The helical residues in this model are colored green. The vertical lines indicate the boundaries of the 8 repeats of model#1 of the I-TASSER server.

Mesothelin is a cell surface protein that is found in normal mesothelium and highly expressed in several cancers including mesotheliomas and ovarian and pancreatic cancers[1,2]. It is produced as a part of the 69 kDa precursor protein[1,3-5]. The furin cleavage of the precursor protein yields two proteins, the N-terminal megakaryocyte potentiating factor (MPF), which is a soluble extra-cellular protein, and the C-terminal 327-residue mesothelin, which is membrane-bound by means of a glycosylphosphatidylinositol (GPI) anchor at the C-terminus of the protein [6]. The sequence of the human mesothelin (from NCBI accession number: NP_005814) is given in Figure 1, which also shows the furin cleavage site and the predicted GPI anchor site. Mesothelin and MPF are useful tumor markers [7,8]. Mesothelin is the target protein of an immunotoxin-based therapy of mesotheliomas and ovarian and pancreatic cancers, of which the phase I clinical trial has been completed [9].

Little is known about the function of mesothelin. It was suggested early on [1] that mesothelin might be involved in adhesion and particularly in adhesion and spread of ovarian cancer cells throughout the mesothelial lining of the peritoneal cavity. However, no phenotype could be detected from mesothelin gene knockout mice [10]. It was found later that mesothelin interacted strongly and specifically with the large glycoprotein MUC16, which is highly expressed in ovarian cancer cells [11,12], and that this interaction was mediated by the N-linked oligosaccharides of MUC16[11]. This interaction presumably plays a major role in the metastasis of ovarian tumors within the peritoneum [11,12]. It was reported recently [13] that mesothelin promoted pancreatic cancer cell proliferation and migration and pancreatic cancer progression, but no molecular mechanism was proposed for these effects.

Mesothelin shares homology with the hypothetical protein MPFL (MPF-like) and with the inner ear proteins otoancorin and stereocilin [14]. These latter two proteins are also GPI-anchored to the membrane of the inner ear sensory and non-sensory epithelial cells and are associated with deafness in people [15,16]. It has been suggested that these proteins interact with the acellular gel that overlies the inner ear epithelium, enabling the inner ear hair cells to detect vibrations in the acellular gel [14-16]. The acellular gel is rich in glycoproteins [17].

We report here a possible three-dimensional (3D) structure of mesothelin based on the results from secondary and tertiary structure prediction programs. We predict that mesothelin has a superhelical structure made of ARM-type helical repeats. Although our main interest is the structure of mesothelin, we performed similar calculations and reached similar conclusions for the structure of the full-length mesothelin precursor protein, as well as for otoancorin and stereocilin. We suggest that all three proteins – mesothelin, otoancorin and stereocilin – function as superhelical lectins that bind the extracellular glycoprotein matrix to the surface of the cell to which they are anchored.

## Results

### Homology search and secondary structure prediction

A BLAST [18] search of non-redundant protein database using human mesothelin precursor protein sequence yielded the hypothetical protein MPFL. A PSI-BLAST [19] run of the same sequence against the Swissprot database converged after three cycles to produce four non-mesothelin hits, which were otoancorins and stereocilins from human and mouse, as expected from a previous report [14]. Three other hits were for mesothelin precursors from mouse, human (a splice variant) and rat. There were no hits from BLAST or PSI-BLAST against Protein Data Bank (PDB) [20] for any of the three proteins mesothelin, stereocilin or otoancorin. A search in Pfam database [21] for mesothelin hits the mesothelin family. No structural information is posted on Pfam for any member of this family.

Results of the secondary structure prediction for human mesothelin sequence from nine different programs [22-26] are shown in Figure 1. They consistently predict a predominantly helical structure, made of small helical segments separated by short non-helical regions. Similar results were obtained for mesothelin precursor, stereocilin, and otoancorin (data not shown). There is one region, residue numbers 291 to 295, which is predicted to be beta strand by all the prediction servers, but this region is presumably either cut away when the protein is modified by the addition of the GPI anchor (see the legend to Figure 1 and the Discussion section) or close to the membrane surface when the protein is anchored to the membrane through the GPI moiety. There are other pockets of beta strand predictions by some, but not by all, prediction servers. Probability scores as calculated by the sam-t02-stride server are high (>0.5) for all the blue colored helical regions in figure 1 and for a couple of beta-patches (the residues 290–295 and 197–201), but those for other beta predictions were all less than 0.5, with average probability of 0.3.

### 3D Structure prediction

Mesothelin and mesothelin precursor sequences were submitted to various 3D structure prediction servers. For the mesothelin precursor, 3D-Jury metaserver [23] produced three hits with Jscore > 50 from three different programs namely INHUB [22], BasD [27] and 3D-PSSM [28]. The hits were 1BK5A, 1WA5B and 1IALA, respectively, which are all superhelical structures with ARM repeats in the 'Armadillo' family in the SCOP protein structure classification database [29]. The predicted region spans the entire length of the precursor (622 amino acids), which includes both MPF and mesothelin. However, the same 3D-Jury metaserver did not produce any hits when only the mesothelin sequence was given. The mesothelin sequence was then submitted directly to the INHUB and 3D-PSSM servers. The highest scoring eight structures from INHUB server were all ARM repeat proteins, 1BK5A being one of them, whereas 3D-PSSM server did not yield any hits with E < 1.0.**(**The mesothelin sequence that was actually used for all calculations reported here inadvertently carried two extra residues, ARG-ARG, at the N-terminus of the sequence. We believe that the presence of these two residues would not significantly affect any of the results reported here, especially in view of the fact that the whole mesothelin precursor is expected to have a non-globular, repetitive structure).

I-TASSER [30] server produced 5 different models for each sequence submitted. All 10 models (5 for mesothelin precursor and 5 for mesothelin) were found to have superhelical structures with ARM-type repeats.

**Figure 2:**
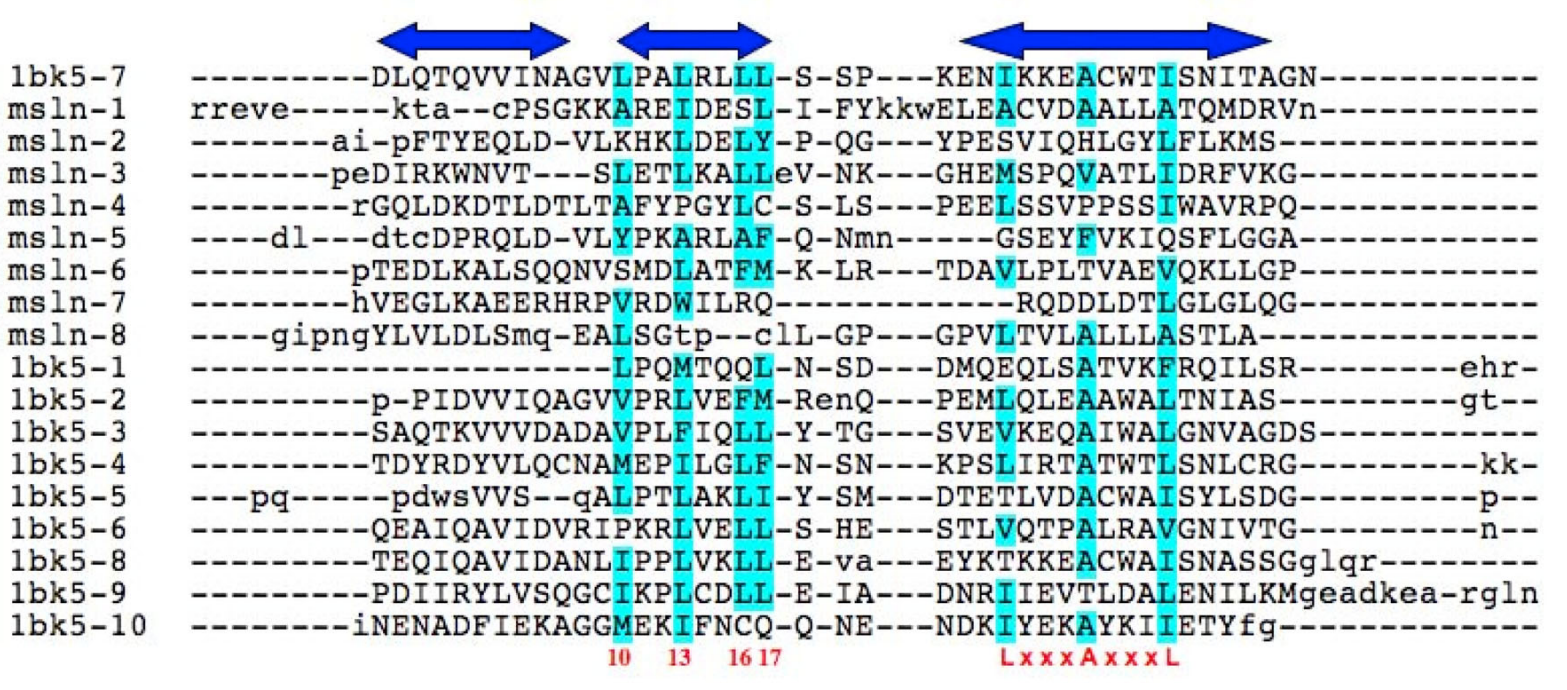
**Multiple alignments of the 10 repeats of 1BK5A (1bk5-1 to 1bk5-10) and the 8 repeats of mesothelin model (msln-1 to msln-8)**. The three double-headed arrows at the top of the Figure indicate the boundaries of the three helical regions of the 7th repeat of 1BK5A. The first two form the outer helices and the third the inner helix. The helix boundaries of other repeats do not always agree with those of the 1BK5A 7^th ^repeat. The residues in the 7^th ^repeat of 1BK5A are shown in the next line in uppercase letters. The residues in all other repeats that follow are in upper or lower case letters depending on whether they align with residues of the 7^th ^repeat of 1BK5A or not, respectively. The columns labelled 10, 13, 16 and 17 at the bottom are the key positions in the outer helix that are hydrophobic in many ARM/HEAT repeats [40]. Similarly LxxxAxxxL at the bottom indicates the conserved LEU and ALA positions in the inner helix, x being any residue. The hydrophobic residues (A, V, I, L, M, F, Y, W) in these 7 columns are highlighted.

**Figure 3:**
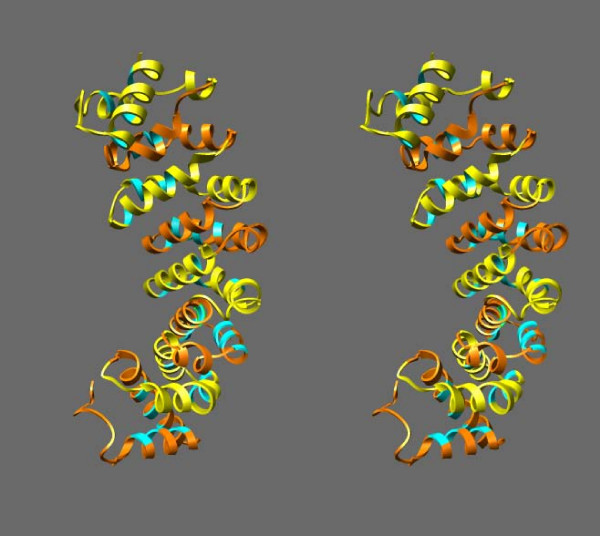
**Stereo pair of the ribbon representation of the 3D structure of model #1 from I-TASSER server**. The chain runs N-terminal to C-terminal from bottom to top in the figure. Repeats 1, 3, 5, and 7 are in orange; repeats 2, 4, 6, and 8 are in yellow; the residues highlighted in Figure 2 are in green. The drawing was made using Chimera [53].

The secondary structures predicted by INHUB server for the INHUB model based on 1BK5A template and those calculated using DSSP [31] software for the first model from I-TASSER server are included in Figure 1. It shows that the secondary structures in these two models largely agree with those predicted by the secondary structure prediction servers. Figure 2 gives multiple structure-based sequence alignments of the 10 ARM repeats of 1BK5A structure [32] and the 8 repeats of mesothelin model#1 from I-TASSER server. (See Methods for the procedure used to make this Figure). The atomic coordinates of model #1 from I-TASSER server for mesothelin can be obtained from the authors. A 3D structural representation of this model is shown in Figure 3.

Other structure prediction servers gave more varied results. Robetta server [24] predicted mesothelin precursor to be made of 4 domains and mesothelin of 2 domains. The program generated 10 models for each of the domains, which were used to build another set of 10 for each of the full chains. All models were made of helices separated by small turns but their overall structures were all different. Most were globular, but a few had the superhelical structure with the ARM/HEAT-type repeats, including one model each for the first 3 domains of mesothelin precursor and another one for the first domain of mesothelin. GenTHREADER [33] server produced no hits for either mesothelin precursor or mesothelin sequence. FFAS [34] predicted (score < -9.5) Leucine-Rich Repeat (LRR) domain structures for mesothelin sequence, which are made mainly of beta strands, turns and coils. These are unlikely to be correct since they are not consistent with the secondary structure prediction results.

Coiled coil is another possible structural type for a predominantly helical protein. In order to see if mesothelin might have a coiled coil structure, we ran two coiled coil detection servers. The COILS server([35] predicted coiled coil fold for mesothelin sequence at three regions, each consisting of only 10–12 residues, one with 0.8 probability and the other two at 0.1 and 0.2 probabilities. Paircoil2 server[36] did not predict any coiled coil fold.

Since the most likely predicted structure is the ARM-repeat type structure, we also ran two servers that detect proteins with repeating motifs in their sequence. The REP server[37] did not identify any repeats for mesothelin. The HHrep server[38] had five hits of potential repeat regions with the highest score 26.7 and with E-values higher than 10^-5^. These are insignificant hits when compared with those for the 1BK5A sequence, in which case one gets hits with scores from 96 to 248 with E-values less than 10^-13^. Therefore, the repeats in mosothelin structure are undetectable using sequence alone and using these tools.

Mesothelin and mesothelin precursor sequences contain regions that are predicted to have disordered structures. The PrDOS server[39] identifies three such regions in the mesothelin precursor: 12 residues at the N-terminus, 3 residues at the C-terminus and an 11-residue segment, REVEKTACPSG, at the furin-cleavage region, which is at the very N-terminal end of the mesothelin sequence (Figure 1). In order to see if the presence of this last potentially disordered region influenced the structure prediction for the mesothelin precursor, both mesothelin and mesothelin precursor sequences were submitted to the 3D-PSSM and INHUB servers with or without the first repeat sequence, msln-1, shown in Figure 2, which includes the REVEKTACPSG sequence. 3D-PSSM gave hits for the mesothelin precursor, which were the ARM-repeat type structures, either with or without the msln-1 sequence. It did not yield any hits for mesothelin with or without the msln-1 sequence. The top 5 hits obtained from the INHUB server, for both mesothelin and mesothelin precursor, were all ARM-repeat proteins, either with or without the msln-1 sequence. Thus, the presence of the middle disordered region apparently did not influence the structure prediction of either protein.

Stereocilin (NCBI accession number: NP_714544.1) with 1775 amino acid residues and otoancorin (DAA00022) with 1153 amino acid residues are about 3 and 2 times the size, respectively, of mesothelin precursor, which has 622 amino acid residues. Both sequences were submitted to the 3D-PSSM server. Since the server can only accept less than 800 residues, stereocilin sequence was submitted in 3 approximately equal parts. Of the total 18 hits (E < 1.0) generated for the three parts, 15 were ARM repeat superhelical structures according to SCOP. The otoancorin was submitted in two equal parts and generated 10 hits (E < 1.0), of which 8 were ARM repeat superhelical proteins.

## Discussion

The mesothelin precursor is most likely to have the superhelical structure with the ARM-type repeats since four different structure prediction programs (INHUB, 3D-PSSM, BasD, and I-TASSER), which employ widely different algorithms, all predict the same type of structure for this protein. This structure is also consistent with the predicted secondary structure. Although other structure prediction programs produced different models, they seem less reliable because the server generated many different structures (Robetta) or the model was inconsistent with the predicted secondary structure (FFAS). Since mesothelin is a part of the mesothelin precursor, mesothelin is also likely to have the same type of structure. Although furin cleavage in general could change the structure of the protein, this seems unlikely for a non-globular, superhelical repeat structure. The secondary structure prediction (Figure 1), the INHUB and I-TASSER server results with the mesothelin sequence alone, and the alignment of the hydrophobic residues among the repeats within the mesothelin part (Figure 2) all support this conclusion.

ARM/HEAT-type superhelical structures are made of tandem repeats of about 50 residue-long helix-turn-helix motifs, of which one helix forms the inner (concave side) and the other the outer (the convex side) helices of the superhelical structure [40]. In the ARM-type repeats, the outer helix is broken into two smaller helices, with a bend in the middle. Typical HEAT-type structures will have ARG and ASP residues interacting between the repeats [40,41]. The ARM repeat proteins lack this ARG and ASP interaction but will often have a GLY and/or PRO residue at or near the bend between the two outer helices. However, there are many ARM/HEAT repeats that do not have either of these features, as can be seen from the aligned sequences in the Pfam family of ARM and HEAT repeats. For example, the recently reported crystal structure of FANCE protein [42] has an ARM/HEAT repeat superhelical structure but without these canonical features. Only the hydrophobic residues are conserved between the repeats in FANCE.

Model#1 for mesothelin from I-TASSER server has a root-mean-square-deviation of 1.9 Å compared to 1BK5A structure as calculated by SHEBA [43] using only the Cα atoms. 1BK5A is an ARM repeat protein with PRO residues in the outer helices. However, neither the I-TASSER models nor the INHUB model of mesothelin built based on 1BK5A structure as template has the regular ARG and ASP interaction between the repeats, nor the GLY or PRO residue between the two outer helices except in one or two repeat (Figure 2). On the other hand, many positions in both the inner and outer helices that are occupied by the hydrophobic residues (residues highlighted in green in Figure 2) in 1BK5A structure are also occupied by similarly hydrophobic residues in the repeats of mesothelin model#1 of I-TASSER server (Figure 2).

The model we present here (Figure 3) is meant to suggest only the type of structure that mesothelin is likely to assume. Although we believe that this is the best structural model for mesothelin at present, the real structure of mesothelin will inevitably be different from that of the model presented. In particular, some or all of the residues of repeat #8 are presumably missing after GPI modification (see below) and it is possible that the remaining residues of the repeats #7 and #8 do not have the typical helix-turn-helix structure of the ARM/HEAT repeats because of their proximity to the cell membrane. This may explain some of the unusual features of repeat #7 of the model structure, which includes a long gap and lacks some conserved hydrophobic residues when compared to the structure of 1BK5A (Figure 2). The very N-terminal region of the mesothelin sequence proper may also have a structure that deviates from the ARM-type repeat since it includes a region predicted to be disordered. Nevertheless, the model was used to suggest a few exposed residues in the N-terminal region of the protein, which might participate in the MUC16 binding. Later experiments showed that the MUC16 binding was indeed significantly affected upon mutation of some of these residues. (Ho et al., accepted for publication in the Journal of Biological Chemistry).

Mesothelin is a glycosylphosphatidylinositol (GPI)-anchored cell-surface protein. The GPI attachment process involves removing all but one residue of the C-terminal signalling sequence and replacing them with the GPI moiety [44]. The exact C-terminal sequence of the GPI modified mesothelin has never been reported, but the region shown in low case italics in Figure 1 has been suggested to be the signalling sequence [1]. We more recently ran a GPI prediction program [45] on the mesothelin precursor sequence. It predicted a smaller region, which is shown in different font color in Figure 1. It turns out that the last repeat (repeat #8) of the model structure is made entirely of the signalling sequence suggested earlier. Therefore, it is probable that GPI-modified mesothelin lacks all or at least a large part of the residues of repeat #8 and that the residues of repeat #7 are close to, and possibly interact with, the GPI anchor and the cell membrane.

The four other models of I-TASSER server are also basically superhelical structures, each one made of eight repeats, each repeat consisting of helices separated by turns. The main differences among them are in the overall twist of the superhelix and in the exact placement of the repeat and helical boundaries, which affect the distribution of hydrophobic and charged residues in the structure. All models lack the ARG-ASP salt bridges between the repeats and the PRO residue in the middle of the two outer helices. We judged that model #1, being most similar to a real superhelical structure, 1BK5, had the most natural superhelical twist of the five models.

This superhelical structure makes it unlikely that mesothelin is an enzyme; well-known superhelical structures of this type function to bind other proteins[37]. We have reported that mesothelin interacts strongly and specifically with the glycoprotein MUC16 and that this interaction appears to be through the carbohydrate moiety of MUC16 [11]. Since mesothelin is not an immunoglobulin and the predicted structure makes it unlikely to be an enzyme, it probably functions as a lectin [46,47]. Although the structure of the carbohydrate-recognition domain (CRD) of lectins is diverse, all CRDs we know are predominantly beta-structures or cysteine-rich domains with little regular structure [48]. Thus, mesothelin appears to be the first example of a lectin made almost entirely of alpha-helices. Another notable feature is that mesothelin and MPF appear to be the first examples of extra-cellular ARM-type repeat proteins. We identified 108 proteins in PDB that have the ARM-type repeats, all of which are intra-cellular.

**Figure 4:**
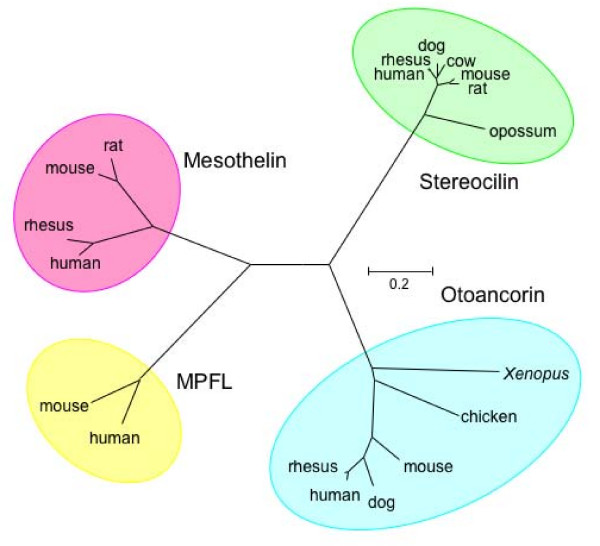
**A phylogenetic tree of mesothelin precursor and related proteins**. The unrooted tree was made using the Neighbor-Joining method [54] implemented in the MEGA3 program [55]. The tree is based on a multiple alignment from the MUSCLE program of the conserved regions of selected mesothelin, MPFL, stereocilin, and otoancorin proteins (see additional file 1 for the alignment). The number 0.2 on the scale bar indicates the number of substitutions per site.

Using BLAST, PSI-BLAST, and the University of California, Santa Cruz Genome Browser database , we could collect a number of sequences that are homologous to mesothelin. Multiple alignment of these sequences using the program MUSCLE [49] and visualized by using the ClustalX program [50] is given as Additional file 1. A phylogenetic tree constructed using this alignment is shown in Figure 4. The MPFLs are relatively close homologues of mesothelin. Stereocilins and otoancorins are more remote homologues, but probably also have the HEAT/ARM type superhelical structures, as predicted by 3D-PSSM. These latter proteins are found attached to the surface of sensory and non-sensory inner ear cells and their defects are associated with deafness [15,16]. They have both been suggested to mediate the attachment of the epithelial and sensory hair cells to the tectorial membrane [14,16], which is the acellular gel that lies over these cells. We suggest that these proteins also function as superhelical lectins, which bind to the polysaccharides of the glycoproteins known to be present in the tectorial membrane [17,51].

## Conclusion

On the basis of the secondary structure prediction from 8 different servers and tertiary structure prediction from 4 different servers, we suggest that mesothelin superfamily of proteins, which includes mesothelin, megakaryocyte potentiating factor, mesothelin precursor, MPFL, stereocilin, and otoancorin, have a superhelical structure made of the ARM/HEAT-like repeats. Partly based on the predicted structure, we propose that all these proteins function as lectins to bind the carbohydrate moiety of glycoproteins.

## Methods

### BLAST, PSI-BLAST and Pfam

BLAST and PSI-BLAST runs were made on the NCBI website  using the default threshold E-value of 10 and inclusion threshold value of 0.005. BLAST was run against PDB, Swissprot and non-redundant sequence databases. Human mesothelin sequence (NCBI accession number NP_005814) was used for all calculations reported in this paper. PSI-BLAST was run on Swissprot database. E< 0.01 was considered as a PSI-BLAST hit. A default E-value of 1.0 was used for Pfam runs. The sequence used for mesothelin for all calculations inadvertently carried two extra residues, ARG-ARG, at the N-terminus of the sequence.

### Secondary Structure prediction

The first entry in Figure 1 is from the INHUB server [22]. Sam-t02-dssp, sam-t02-stride and profsec programs are from the 3D-Jury metaserver [23]. Psipred, sam, and jufo programs are from the Robetta server [24]. Results from two more servers, DSC [25] and APSSP2 [26], are also included. The last entry in Figure 1, labelled I-TASSER, was calculated using the DSSP program on the I-TASSER model#1.

### Submission of sequences to 3D structure prediction servers

The mesothelin precursor and mesothelin sequences were separately submitted to 3D-Jury , GenTHREADER , INHUB , 3D-PSSM  and I-TASSER servers . In addition, mesothelin alone was submitted to FFAS . For 3D-Jury results, jscore > 50 was considered as a hit. For the 3D-PSSM results, we considered all hits with E< 1.0, since E-values below 0.05 were suggested to be highly confident and E-values up to 1.0 as worthy of attention. According to the instructions for FFAS, predictions with scores lower than -9.5 contain <3% of false positives and we chose this as the cutoff value. INHUB and I-TASSER did not have suggested threshold values for their results.

### Submitting mesothelin precursor sequence to servers that predict disordered regions and coiled coil motifs and internal repeats

Sequence of mesothelin precursor was submitted to PrDOS server  that predicts the disordered regions of a protein from its amino acid sequence and also to two servers, Paircoil2  and COILS  both of which predict coiled coil fold from sequence. Sequence of mesothelin precursor was also submitted to REP server , which searches for repeats similar to those in its database containing ARM, HEAT, ANKYRIN and other protein repeats. Similarly, sequence of the mesothelin precursor was submitted to HHrep server  which identifies internal repeats within a given protein sequence.

### Multiple alignment of the repeats of mesothelin

The multiple alignments shown in Figure 2 include the 8 repeats of mesothelin of I-TASSER model#1, along with the 10 ARM repeats of 1BK5A. The 10 structural repeats of 1BK5A were derived using the repeat boundaries reported by the authors [32]. Similar repeats for mesothelin were derived for the I-TASSER model#1 after this model was structurally superposed to 1BK5A. Each of the 9 repeats of 1BK5A and 8 repeats of mesothelin were then aligned pairwise to the 7^th ^repeat of 1BK5A using SHEBA. The sequence alignments from these pairwise structural alignments were read out using the program SE[52] and collected together in a consistent manner using the 7^th ^repeat of 1BK5A as the anchor sequence.

### Submission of stereocilin and otoancorin to 3D-PSSM server

Stereocilin and otoancorin sequences were submitted to the 3D-PSSM server in pieces because there is a limit of 600 residues that one can submit to the 3D-PSSM server. The stereocilin sequence was broken into 3: the N-terminal part (residues 1–593), the middle part (594–1191) and the C-terminal part (1192–1775); the otoancorin sequence was broken into 2: the N-terminal part (1–560) and the C-terminal part (561–1137).

## List of abbreviations

CRD: carbohydrate-recognition domain; GPI: glycosylphosphatidylinositol; ALA: alanine; ARG: arginine; ASP: aspartate; GLY: glycine; LEU: leucine; PRO: proline

## Competing interests

The authors declare that they have no competing interests.

## Authors' contributions

BKS ran most of the programs, YH found the homologs of mesothelin and made multiple alignments of them and the phylogenetic tree, MP, IP and BL generated the idea of this research and contributed through editing the manuscript and taking part in the discussions, BL directed the research, and BKS and BL were primarily responsible for writing the manuscript. All authors read and approved the manuscript.

## Supplementary Material

Additional file 1 **Supplemental figure 1, Multiple alignments of the conserved regions of the 19 homologues of mesothelin precursor using MUSCLE**. These alignments were used to construct the phylogenetic tree in Figure 4. The protein sequences include human, mouse and rat mesothelin precursors (accession numbers NP_005814, NP_061345, and NP_113846, respectively); mouse MPFL (MPF-like, also known as BC052484, accession number NP_808490); human, mouse, dog, and cow stereocilins (accession numbers NP_714544, NP_536707, XP_535452, and XP_606859, respectively); human, mouse, and *Xenopus *otoancorins (accession numbers NP_653273, NP_647471, and AAH79797, respectively); and predicted sequences of mesothelin precursor from rhesus macaque; MPFL from human; stereocilin from rhesus macaque, rat, and opossum; and otoancorin from rhesus macaque, dog, and chicken. The aligned regions of the representative proteins are: human mesothelin precursor, 68–502; mouse MPFL, 63–510; human stereocilin, 1194–1666; and human otoancorin, 570–1020. Coloring and the quality curve are as described at . '*' indicates fully conserved column, ':' strongly conserved and '.' weakly conserved. Human mesothelin sequence starts at position 273. The histogram below the alignment is the alignment quality curve. Click here for file
