# Supplementary material for: Mesothelin, Stereocilin, and Otoancorin are predicted to have superhelical structures with ARM-type repeats
Source: BMC Struct Biol. 2009 Jan 7;9:1. doi: 10.1186/1472-6807-9-1 (PMC2628672; doi:10.1186/1472-6807-9-1)

```
MSLN_human    GFPCAEVSGLS-TERVRELAVALAQKNVKLSTEQLRCLA------HRLSEPPE--------------DLDALPLDLLLFLNPDAFSGPQACTRFFSRITKANVDLLPRGAPERQRLLPAALACWGVRGSLLSEADVRALGGLACDLPGRFV--AESAEVLLPRLVS
MSLN_rhesus   GFTCVEVSGLS-TELVQELAVALGQKNVKLSAEQLRCLA------HQLSEPPE--------------DLDALPLDLLLFLNPDAFSGPQACTHFFSRVAKANVDLLPRGAPERQRLLPGALTCWGVRGSLLSEADVRALGGLACDLPGRFV--AESAEVVLPRLVR
Msln_mouse    GLTCEEVSDLS-MEQAKGLAMAVRQKNITLRGHQLRCLARRL---PRHLTDEE--------------LNALPLDLLLFLNPAMFPGQQACAHFFSLISKANVDVLPRRSLERQRLLMEALKCQGVYGFQVSEADVRALGGLACDLPGKFV--ARSSEVLLPWLAG
Msln_rat      GLTCDEVSGLS-MGHAKELAMAVRQKNIVLQVHQLRCLARRL---PKHLTNEE--------------LDALPLDLLLFLNPAMFPGQQACAHFFSLISKANVNVLPRRSLERQRLLTGALKCQGVYGFQVSETDARALGGLACDLPGEFV--AKSSEVLLPWLAR
MPFL_human    GFWCQPASQLP-RDQLSALIQRLALLQVPLQAWQLSCLANLA---SRCGLQDD--------------FTLHPPNLLLFYNLSQVREAD-CRAFIRRAAQGDVELLSHLPDQRVALWRAAVACLGVARLRLSASDQQLLGALVCDMDASSI--GAADPHMLENLRR
Mpfl_mouse    NFWCLPASQLP-REQLSSLIRSLASQRVALKAWQLSCLANLA---AQLGLQDD--------------FEFHPPNLLLFYDLSQVGDTN-CRAFIHHAAQGDTELLTNLPNQRVALQRTALACLGGPHLQLSASDLWLLGVLVCDMEAAQI--VTADPSVLRNLLR
OTOA_human    GVTCSHIDAMS-TDFFLAHFQDFQNNFALLSPYQVNCLAWKYWEVSRLSMPPF--------------LLAALPARYLASVPASQ------CVPFLISLGKSWLDSLVLDSHKKTSVLRKVQQCLD--DSIADEYTVDIMGNLLCHLPAAIIDRGISPRAWATALHG
OTOA_rhesus   GVTCSHIDAMS-TDFFLAHFQDFQNNFALLSPYQVNCLAWKYWEVSRLSMPPF--------------LLAALPARYLASVPASQ------CVPFLISLGKSWLDSLVLDSHKKTSVLRKVQQCLD--NSIADEYTVDIMGNLLCHLPAAIIDRGISPRAWATALHG
Otoa_mouse    GVPCSHIDAMS-DHLFLALFQYFDNNFSLLSPDQVNCLAWKYWEVSRSSMPPF--------------LLATLPSRFLSSIPPSR------CVRFLISLGKRRLETLVLDSDKRSVVVRKVQQCLD--GVIADEYTVDIVGHLLCHLPASFIERGISPRAWAAALHG
OTOA_dog      GMTCSHIDAMS-ADSFLALHFHYFENNLSLLSPYQVNCLAWKYWEVSRSSMPPF--------------LLAALPAHHLASVPASQ------CVPFLISLGKNQLDSLVLDSHKKNSVLRKVQQCLN--NSIADEFAVDIVGKLLCHLPAAIIHSGISARAWATALHG
OTOA_chicken  GVTCQLIESMG-TDSFLNHFKVFENNLHLLSPYQINCLAWKFWEVSNASIPPF--------------FFLVLPTEYLEYISGPL------CVPFIESLGKTEVDLLSPSFHKKETVLQKVQECLN--GSITDEYDVDLLGNLICHLPPAFLHARMSLKATATALHQ
OTOA_Xenopus  GLTCEHIDSMD-KPSFLNHYKLLENSLSLLTPYQIHCLAWKYWKVSEATIPVF--------------LLAVLPSERFASYSIP------CGSLLISLGKAELSYLVLNASKKERIMNKVDQCLN--SSLADAYQLDMIGNLICHLSPKIIKSGISTDVIAAAINH
STRC_human    GMSCEFLQQINSMVDFLEVVHMIYQLPTRVRGSLRACI-WAELQ-RRMAMPEPEWTTVGPELNGLDSKLLLDLPIQLMDRLSNES------IMLVVELVQRAPEQLLALTPLHQAALAERALQNLAPKETPVSGEVLETLGPLVGFLGTEST-RQIPLQILLSHLSQ
STRC_rhesus   GMSCEFLQQINSMVDFLEVVHMIYQLPTRVRGSLRACI-WAELQ-RRMAMPEPEWTTLGPELNGLDSKLLLDLPIQLMDRLSNES------IMLVVELVQRAPEQLLALTPLHQAALAERALQNLAPKETPLSGEVLETLGPLVGFLGIEST-RQIPLQILLSHLSQ
Strc_mouse    GMTCEFLQQISSMVDFLDVVHMLYQLPTGVRESLRACI-WTELQ-RRMTMPEPELTTLGPELSELDTKLLLDLPIQLMDRLSNDS------IMLVVEMVQGAPEQLLALTPLHQTALAERALKNLAPKETPISKEVLETLGPLVGFLGIEST-RRIPLPILLSHLSQ
Strc_rat      GMSCEFLQQISSMVDFLDVVHMLYQLPIGVRESLRACI-WAELQ-RRMAMPEPELTTLGPELSELDTKLLLDLPIQLMDRLSNDS------VMLVVELVQGAPEQLLALTPLHQAALAERALKNLAPKETPISQEVLETLGPLVGFLGIDST-RRIPLPILLSHLSQ
STRC_dog      GMSCEFLQQINLMADFLEVVHMIYQLPTGVRGSLRACI-WAELQ-RKMTMPEPELATLGSELSGLDTKLLLDLSPIYLMDRLSNES------IMLMVELVRGAPEQLLALTPLHRVALAERALQNLAPKETTVSREVLETLGPLVGFLGIEST-RRIPLPILLAHLNQ
STRC_cow      GMSCEFLQQINWMADFLEVVHMIYQLPTGVRGSLRTCI-WAELQ-RRMAMPEPELATLGPELSGLDTKLLLDLPVQLMDRLSNES------IMLVVELVRGAPEQLLALTPLHRAALAERALQNLAPKETTVSREVLETLGPLVGFLGIEST-RRIPLQILLAHLSQ
STRC_opossum  GMTCEWLQQFSWRSDFLKVVVMLYQLPNGVRGSLRACI-WEELQ-RKMEMSELGLGGLGPDLNGLEQELLFQLPIQLIDRLSNDS------ILLMLELIRGDPELFLVLPPIRREALAERALRSLAPQDIPLSGEVLEALGPLVGSLGTESV-GRILPQTLLAHLSR
ruler         1.....10.....20.....30.....40.....50.....60.....70.....80.....90.....100.....110.....120.....130.....140.....150.....160.....
```

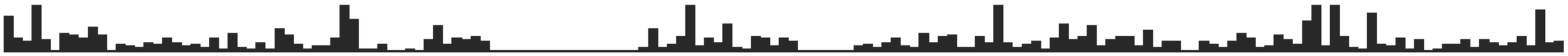

```
MSLN_human    ----CPGPLDQDQQEAARAALQGGGPPYGPPSTWSVSTMDALRGLLPVLGQPIIRSIP-QGIVAAWRQR---------SSRDPSW------RQPERTILRPRFRREVEKTACPSGKKAREIDES------L--IFYKKWELEACVDAALLATQMDRVNAIP-FTYEQ
MSLN_rhesus   ----CLGPLDQDQQEAARAALQRGGPPYGPPSTWSISTLDDLQSLLPVLGQPVIHSIP-QGILAAWRQR---------SSRDPSW------QQPEQTVLRLRFRRDVERTTCPPEKEVHEIDES------L--IFYKKRELEACVDPALLAAQMDRVDAIP-FTYEQ
Msln_mouse    ----CQGPLDQSQEKAVREVLRSGRTQYGPPSKWSVSTLDALQSLLVAVLDESIVQSIP-KDVKAEWLQH---------ISRDPSR------LGSKLTVIHPRFRRDAEQKACPPGKEPYKVDED------L--IFYQNWELEACVDGTMLARQMDRVNEIP-FTYEQ
Msln_rat      ----CGGPLDQGQAKAVREVLRSGRAPYGPPSTWSVSTLDALQGLLVVLDESIVHSIP-KDVITEWLQG---------ISREPSR------LGSKWTVTHPRFRRDTEQKACPPGKEPNVVDEN------L--IFYQNWELEACVDGTLLAGQMDLVNEIP-FTYEQ
MPFL_human    ----CPR-LTAAQRIALNSLLAGGKTSLGPPGSWTLEGLQALGPLATYISPHLWAQVQ-EAVGLGFFRSVVASCQVGRLGQREARCFVTSFLESKTKPVSSRPRLSTGGPCVXGNITAATLRDD------LFLVHYDCAELESCLDGCILRTNLDTLLQHL-LPTEC
Mpfl_mouse    ----CPR-LTVMQTAALNTLLASGKTQIGPPGSWNLQGLQALGLLATYISPHLWEKVQ-EAVGLDFFRSVVAACRAGQLSRHDARRFVDNFLESKATSVSSRPKRRTGRSCVRGNITAATLHDD------LFLVHYDCTQLESCLGTRVLRANLDPLLQHP-LPAEC
OTOA_human    LRD-CPD-LNPEQKAAVRLKLL-GQ--YGLPQHWTAETTKDLGPFLVLFSGDELSSIA-TKF-PEILLQAASKMARTLPTKEFLWAVFQSVRNSSDKIPSYDPMPGCHGVVAPSSDDIFKLAEA------N--ACWALEDLR-CMEEDTFIRTVELLGAVQGFSRPQ
OTOA_rhesus   LRD-CPD-LNPKQKAAVRLKLL-GQ--YGLPQHWTAETTKDLGPFLVLFSGDELSSIA-TKF-PEILLQAASKMARTLPPKEFLWAVFGSVRNSSDKIPSSDPMPGCHGVAAPSSDDIFKLAEA------N--ACWALKDLR-CMEEDTFIRTVELLGAVQGFSRPQ
Otoa_mouse    LRS-CTA-LSSEQKAAVRVRLL-EQ--WGPPENWTAETTKDLAPFLAFFSGDELHTVA-TKF-PEILQQTASKMVGVLLPKEFLWAVFESVQNSSNESPSFDPTFGCHGVVTPSSDDIFKLAEA------N--ACWDPEVLL-CMEEDTFIRNVELLGAVKGFSRAQ
OTOA_dog      LRD-CPG-LSPEQKAAVRLRLL-EQ--YGLSRNWTAETTMKDLGPFLVLFSGDELSSVA-TKF-PEILQQTSFKIAGTLPPPEFLWAVFESVWNSSERNPSSHPSPGCHGVMAPSSSDIFKLAEA------N--ACWAPEDLL-CMEEHTFIRSVELLGAVRAFSLPQ
OTOA_chicken  FKL-CRQ-LSHEQKTEIKYKLL-EL--YGSPKNWTAETTLDVGPFIAQLSKGELNVLA-EKF-PDIILRIAKTIGPPSSAEELLSTVFESVFNATASMEPHLTTPDCLGTRAPSSDEIIKLAEA------N--VYWSAQELK-CMDAGTFDKNVELLGTVSGFNNSQ
OTOA_Xenopus  LKS-CRN-LSPSQNTEIKYRLI-EL--YGHPSNWTSETVQDMAPFWNLLSKDEFLDIL-KKF-QNTVLQMVSEPPGIPLSKGILSVVFDAVRLLGVNVPVANHTADCTGIMGPSADDITRLMDA------N--AFWSSAELQ-CIDIDTFTKIVHILGAIQSFNESQ
STRC_human    LQGFC---LGETFATELGWLLL-QESVLGKPELWSQDEVEQAGRLVFTLSTEAISLIPREALGPETLERL--------LEKQQSWEQSRVGQLCREPQLAAKKAALVAGVVRPAAEDLPEPVPNCADVRGTFPAAWSATQIA-EMELSDFEDCLTLFAGDPGLGPEE
STRC_rhesus   LQGFC---LGETFATELGWLLL-QESVLGKPELWSQDEVEQAGRLVFTLSTEAISLISREALGPETLERL--------LEKQQSWEQSRVGQLCRGPQLAAKKAALVAGVVRPAAEDLPEPVPNCADVRGTFPAAWSATQIA-EMELSDFEDCLTLFAGDPGLGPEE
Strc_mouse    LQGFC---LGETFATELGWLLL-QEPVLGKPELWSQDEVEQAGRLVFTLSAEAISSIPREALGPETLERL--------LGKHQSWEQSRVGHLCGESQLAHKKAALVAGIVHPAAEGLQEPVPNCADIRGTFPAAWSATQIS-EMELSDFEDCLSLFAGDPGLGPEE
Strc_rat      LQGFC---LGETFATELGWLLL-QEPVLGKPELWSRDEIEQAGRLVFTLSAEAISLIPREALGPETLERL--------LGKQQSWEQSRVGHLCGKSQLAHKKTALVAGIVHPAVEGLQDPVPNCADIRGTFPAAWSATQIA-EMELSDFEDCLSLFAGDPGLGPEE
STRC_dog      LQGFC---LGEPFATELGWLLS-QEPILGKPELWSEGEVEQAGRLVLTLSTEAISLIPREALGPETLERL--------LEKQQSWEQSRVGQLCGTPQLAPKKAALVAGVVRPTAEDLSEPVPNCADVRGTFPAAWSATQIA-EMELSDFEDCLALFAGDPGLGPEE
STRC_cow      LQGFC---LGGLFATELGWLLL-QEPVLGKPELWSQDEVEQAGRLIFTLSPEAISLIPREALGPETLERL--------LEKQQSWEQSRAGQLCAGPQLAPKKAALVAGLVRPAAENLPEPVPNCADVRGTFPSAWSAAQIA-DMELLDFEDCLALFAGDPGLGPEK
STRC_opossum  LQNFC---LGEPPFVTELGCLLL-HESAFGRPELWSRDEVEQAGRLVLTLSSQAILLLPREALGPETLERL--------LERQQNWEQSRVGQLCGGPVLGPRKAALVTGAVQAEDENEPDPVPTCADIRGTFPAAWSAAQIT-EMGLSDFKDCLGLFAKDPSLKPAE
ruler         170.....180.....190.....200.....210.....220.....230.....240.....250.....260.....270.....280.....290.....300.....310.....320.....330....
```

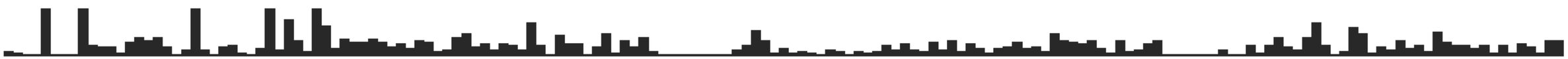

```
MSLN_human    LDVLKHKLDELY--PQGYPESVIQHLGYLFLKMSPEDIRKWNVTSLETLKALLEVNKGHEMSPQVATLIDRFVKGRGQLDKDTLDTLTAFYPGY--LCSLSPEELSSVPPSSIWAV----RPQDLDTCDPRQLDVLYPKARLA--FQNMN--GSEYFVKIQSFLGG
MSLN_rhesus   LDVLKHKLDELY--PQGYPESVIRHLGHLFLKMSPEDIRKWNVTSLETLKALLKVSKGHEMSAQVATLIDRVVVGRGQLDKDTVDTLTAFCPGC--LCSLSPERLSSVPPSVIGAV----RPQDLDTCGPRQLDVLYPKARLA--FQNMS--GSEYFVKIRPFLGG
Msln_mouse    LSIFKHKLDKTY--PQGYPESLIQQLGHFFRYVSPEDIHQWNVTSPDTVKTLLKVSKGQKMNAQAIALVACYLRGGGQLDEDMVKALGDIPLSY--LCDFSPQDLHSVPSSVMWLV----GPQDLDKCSQRHLGLLYQKACSA--FQNVS--GLEYFEKIKTFLGG
Msln_rat      LSIFKHKLDKTY--PQGYPESLIKQLGHFFRYVSPEDIRQWNVTSPDTVNTLLKVSKGQKMDAQVIALVACYLRGGGKLDEDIVKALDNIPLSY--LCDFSPQDLHAIPSSVMWLV----GLHDLDKCSQRHLGILYQKACSA--FQNVS--GLEYFEKIRTFLGG
MPFL_human    QHVVKAKLAQIY--PQGLPEDQLRLITSLVYLYSRTEIGQWSITSQDTVMALLASDVA--LENQTEAVLQKFLEHNGTVSGA-L----LLAIGGTRLCWMSPHQIQTIHPQELRLA----GALDLSSCPQSRKDVLYTKAHET--FGSSGTLA-AYYRLMRPYLGG
Mpfl_mouse    QRVVKAKLTQIY--PHGIPEDQLHLIPSLVYLYSLAEIGQWNITSGDTVMILLASD-A-ALDNQTEAVLQRFLDHNGKVTGA-L----LVAIGGSRLCWMSLKQLQFIQPSEFRLA----GAPDISPCPQSRKDALFVKAHEV--FRNTSNVG-EYYYLIRPYLGG
OTOA_human    LMTLKEKAIQVWDMPSYWREHHIVSLGRIALALNESELEQLDLSSIDTVASLSWQT-E-WTPGQAESILQGYLDDSGYSIQD-LKSFHLVGLGAT-LCAINITEIPLIKISEFRVVVARIGTLL---CSTHVLAEFKRKAEVV--FGDPTEWTSSVLQELGTIAAG
OTOA_rhesus   LMTLKEKAIQVWDMPSYWREHHIASLGRIALALNESELEQLNLSSIDTVAFLTQQT-E-WSPRQAESILQGFLDDSGYNIQD-LKSFHLVGLGAT-LCAINITEIPLIKISEFRVVVARIGTLL---CSTHVLAEFKRKAEVV--FGDPTEWTSPVLQELGTIAAG
Otoa_mouse    LMALKEKAIQVWDLPSRWKEHHIVSLGRIALALSESELEQLDLSSIDTVASLGQQT-E-WTPGQAKSILQAFLEDSGYGIQD-LKSFHLVGFGPT-LCAMDPTEIQLIKTSEFRAVVARIGTLF---CSTPVLAGFKKKAEVV--FGRPTEWTSSILQELGTIAAG
OTOA_dog      LVTLKEKAVQVWDMPSYWKEHHITSLGRIALALNESELEQLDLSSIDTVVSLSQQT-E-WTPGQAKSILQGFLEDSGYSVQD-LKSFHLVGLGTT-LCAMNITEISLIKISEFRVVMARIGTLL---CSIHILAEFKRKAEAV--FGHPTKWSSSVLQELGTIAAG
OTOA_chicken  LMALKEKAKQVWGSLLGWKSYHIVSLGHIALALTENEIEELDLHSIDTVSVLSQQA-EEWTLAQARSILQSFLEDSGQTMST-LKSFDLVGLGAI-LCALNSTEIMSIRTAEFSAAVARIGLLL---CSTPVLKQFKKITESV--FGTATSWNGSVLQEIGTIAGG
OTOA_Xenopus  LSVLKEKAKEAWGDLPSWKSYHITSLGHIATALSKQEIDELDLSNIDAVSALGQQA-E-WSSVQVKAILHGFLNDSAKAPSD-LKSYHLAGLAGT-LCAANAKQIEEIQTSEFRAVISRIGSLP---CEMSVLQAFKNKAEVV--YGKSDQWSHFIINDIGLIAAG
STRC_human    LRAAMGKAKQLWGPPRGFRPEQILQLGRLLIGLGDRELQEELILVDWGVLSTLGQID-G-WSTTQLRIVVSSFLRQSGRHVSH-LDFVHLTALGYT-LCGLRPEELQHISSWEFSQAALFLGTLHLQ-CSEEQLEVLAHLLVLPGGFGPISNWGPEIFTEIGTIAAG
STRC_rhesus   LRAAMGKAKQLWGPPRGFRPEQILQLGRLLIGLGDRELQEELILVDWGVLSTLGQID-G-WSSTQLRVVVSSFLRQSGRHVSH-LDFIHLTALGYT-LCGLRPEELQHISSWEFSQAALFLGTLHLQ-CSEEQLEVLAHLLVLPGGFGPISNWGPEIFTEIGTIAAG
Strc_mouse    LRAAMGKAKQLWGPPRGFRPEQILQLGRLLIGLGERELQEELTLVDWGVLSSLGQID-G-WSSMQLRAVVSSFLRQSGRHVSH-LDFIYLTALGYT-LCGLRPEELQHISSWEFSQAALFLGSLHLP-CSEEQLEVLAYLLVLPGGFGPVSNWGPEIFTEIGTIAAG
Strc_rat      LQAAMGKAKQLWGPPRGFRPEQILQLGRLLIGLGERELQEELTLVDWGVLSSLGQID-G-WSSVQLRAVVSSFLRQSGRHVSH-LDFIYLTALGYT-LCGLRPEELQHISSWEFSQAALFLGSLHLP-CSEAQLEALAYLLVLPGGFGPVSNWGPEIFTEIGTIAAG
STRC_dog      LRAAMGKAKQLWGPPRGFRPEQILQLSRLLIGLGERELQEELILVDWGVLSTLGQID-G-WSSIQLRVVVSSFLRQSGRHVSH-LDFLHLTALGYT-LCGLRPEELQHISSWEFSQAALFLGNLHLQ-CSEEQLEVLAQLLVLPGGFGPVSNWGPEIFTEIGTIAAG
STRC_cow      LRAAMGKAKQLWGPPRGFRPEQILQLGRLLIGLGERELQEELSLVDWGVLSTLGQID-G-WSSSQLRVVVSSFLRQSGRHVSH-LDFLHLTALGYA-LCGLRPEELQHISSWEFSQAALFLGNLHLP-CSEEQLEILAQLLVLPGGFGPVSNWGPEIFTEIGTIAAG
STRC_opossum  LQAAMSKAKELWGPPQRLHPEQILQLGRLLIGLGEHELQEELILVDWAVLGALGQLE-G-WSKIQLQIIVSSFLHQSGRHVRN-LDFIHLTALGHA-ICGLRPDEIQQINSREFSKAVLFLGFLPLQ-CSEEQLEALAHLLVLPGGFGLVSDWGPEIFTEVGSVAAG
ruler         ...340.....350.....360.....370.....380.....390.....400.....410.....420.....430.....440.....450.....460.....470.....480.....490.....500
```

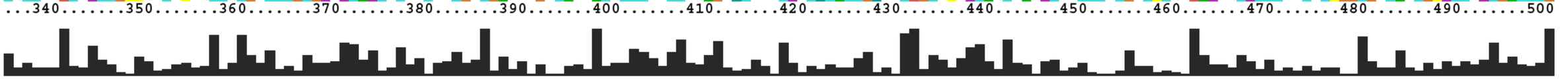

Supplement: Additional file 1 — Supplemental figure 1, Multiple alignments of the conserved regions of the 19 homologues of mesothelin precursor using MUSCLE. These alignments were used to construct the phylogenetic tree in Figure 4. The protein sequences include human, mouse and rat mesothelin precursors (accession numbers NP_005814, NP_061345, and NP_113846, respectively); mouse MPFL (MPF-like, also known as BC052484, accession number NP_808490); human, mouse, dog, and cow stereocilins (accession numbers NP_714544, NP_536707, XP_535452, and XP_606859, respectively); human, mouse, and Xenopus otoancorins (accession numbers NP_653273, NP_647471, and AAH79797, respectively); and predicted sequences of mesothelin precursor from rhesus macaque; MPFL from human; stereocilin from rhesus macaque, rat, and opossum; and otoancorin from rhesus macaque, dog, and chicken. The aligned regions of the representative proteins are: human mesothelin precursor, 68–502; mouse MPFL, 63–510; human stereocilin, 1194–1666; and human otoancorin, 570–1020. Coloring and the quality curve are as described at . '*' indicates fully conserved column, ':' strongly conserved and '.' weakly conserved. Human mesothelin sequence starts at position 273. The histogram below the alignment is the alignment quality curve. [file 1472-6807-9-1-S1.pdf]
